# Crystallization Thermodynamics of α-Lactose Monohydrate in Different Solvents

**DOI:** 10.3390/pharmaceutics14091774

**Published:** 2022-08-25

**Authors:** Youliang Guan, Zujin Yang, Kui Wu, Hongbing Ji

**Affiliations:** 1 School of Chemical Engineering and Technology, Sun Yat-sen University, Zhuhai 519082, China; 2 School of Chemistry and Chemical Engineering, Jinggangshan University, Ji’an 343009, China; 3 School of Chemistry, Sun Yat-sen University, Guangzhou 510275, China

**Keywords:** α-lactose monohydrate, solubility models, solvent effect, dissolution thermodynamics

## Abstract

It is common to find that some of the lactose in dairy powders and pharmaceutical tablets is present in the unstable amorphous state. Therefore, their crystallization thermodynamics in different solvents are particularly important. In this paper, the solubility of α-lactose monohydrate (α-LM) in 15 mono-solvents such as ethanol, isopropanol, methanol, 1-propanol, 1-butanol, 2-butanol, isobutanol, 1-pentanol, isoamylol, 1-hexanol, 1-heptanol, 1-octanol, propanoic acid, acetonitrile, and cyclohexanone was evaluated by using the gravimetric method in the temperature ranges from 274.05 K to 323.05 K at constant pressure (1 atm). In the given temperature range, the solubility of α-LM in these solvents increased with the rising of temperature, the highest solubility of α-LM was found in methanol (2.37 × 10^4^), and the lowest was found in 1-hexanol (0.80 × 10^5^). In addition, the increase of α-LM solubility in isopropanol was the largest. The sequence at 298.15 K was: methanol > 1-butanol > isopropanol > ethanol > 1-propanol > 1-heptanol > isobutanol > propionic acid > 1-pentanol > 1-octanol > acetonitrile > isoamylol > 2-butanol > cyclohexanone > 1-hexanol. Solvent effect analysis shows that the properties of α-LM are more important than those of solvents. The Apelblat equation, λh equation, Wilson model, and NRTL model were used to correlate the experimental values. The root-mean-square deviation (*RMSD*) and relative average deviation (*RAD*) of all models were less than 2.68 × 10^−2^ and 1.41 × 10^−6^, respectively, implying that the fitted values of four thermodynamic models all agreed well with the experimental values. Moreover, the thermodynamic properties of the dissolution process (i.e., dissolution Gibbs free energy (Δ*_dis_G*), molar enthalpy (Δ*_dis_H*), and molar entropy (Δ*_dis_S*)) for α-LM in selected solvents were determined. The results indicate that Δ*_dis_H*/(J/mol) (from 0.2551 to 6.0575) and Δ*_dis_S*/(J/mol/K) (from 0.0010 to 0.0207) of α-LM in these solvents are all positive, and the values of Δ*_dis_H* and Δ*_dis_S.* Δ*_dis_G*/(J/mol) (from −0.0184 to −0.6380) are all negative. The values were observed to decrease with rising temperatures, implying that α-LM dissolution is an endothermic, entropy-driven, and spontaneous process. The solid–liquid equilibrium data and dissolution thermodynamics of α-LM were obtained, which provide a basis for industrial production.

## 1. Introduction

**Figure 1:**
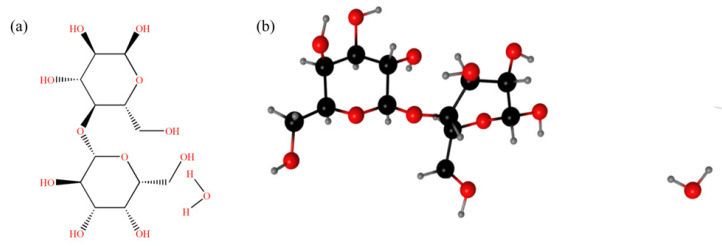
Structure of α-LM: (**a**) chemical molecular; (**b**) ball and stick model.

Lactose (4-O-β-D-galactopyranosyl-D-glucopyranose), a by-product of the milk industry, is produced from the solution crystallization of whey [1,2]. Due to the different orientation of -OH groups in glucose unit, lactose molecule has two different isomers (i.e., α and β anomers) [3]. The α-lactose monohydrate (C_12_H_22_O_11_·H_2_O, molar mass 360.31 g·mol^−1^, CAS No. 5989-81-1, abbreviated as α-LM, the chemical structure shown in Figure 1), the most stable form of lactose, is widely used as sweetener, stabilizer, and excipient in food and pharmaceutical excipient because of its excellent texture, taste, and adhesion [4,5,6]. However, α-lactose products usually exist as a mixture of α and β-lactose in the aqueous solution with a ratio of 60:40 [7]. Altamimi et al. used a new H^1^-NMR to analyze a group of 19 commercial lactose samples to establish a library, implying the isomer content of a large number of lactose products, because a change of more than 10% in the isomer content of the α-lactose monohydrate sample may affect the bioavailability of the final preparation [8]. Therefore, the purity of α-LM in the production process will significantly affect its functions, so it is necessary to purify the food and drug additive, and the solubility of the additive in various pure solvents can provide a theoretical basis for the food and drug crystallization process. Different methods such as anti-solvent crystallization [4,5], micro-fluidic spray drying [9], ultrasonic crystallization [10,11], and solvated crystallization [12] have been used to improve the yield and the desired shape, size, and polymorphic form of α-LM crystals. Industrial production of α-LM is an energy-intensive process. Optimized solvents can recover α-LM from the mixture of α- and β-lactose, which can save stringent energy and help regulate the nucleation, growth, and polymorphism of α-LM crystals. López-Pablos et al. proposed a method to prepare pure anhydrous β-lactose (β-L) by static reaction of α-LM in alkaline alcohol solution under the condition of controlling temperature [13]. Therefore, obtaining the solubility data of α-LM in different solvents helps choose the appropriate crystallization solvent and production process. Majd et al. measured the solubility of lactose in 70–90% alcohol-aqueous solution, which guided the recovery of lactose crystals [14]. Choscz et al. discussed the effects of different whey salts and mixed salts on the solubility of lactose in aqueous solution (20–50 °C), and proposed a semi-predictive modeling method based on EPC-SAFT model [15]. Machado et al. obtained the solubility of α-lactose in water–ethanol mixed solvents (25, 40 and 60 °C, with concentrations ranging from 0 to 100 wt.% water), and correlated and predicted it using the UNIQUAC model [16]. But there have been few relevant reports on the solubility distribution of α-LM in different pure solvents, solvent effect, and thermodynamic properties of the dissolution process in previous literature.

Alcohols, ketones, and hydrocarbons are commonly used organic solvents in industrial operations (including chemical reactions, preparation and separation). From various lengths of the carbon chain to different types of isomerism, multiple homologous alcohols exist and different alcohols show different hydrophilic and hydrophobic propensities. Most organic solvents are usually volatile, and therefore solution crystallization does not need too-high temperatures; solubility data in a temperature range 0–60 °C is enough for daily use. Thus, the purpose of this work was to accurately determine the solid–liquid equilibrium data of α-LM in 15 pure solvents, including methanol, ethanol, 1-propanol, isopropanol, 1-butanol, 2-butanol, isobutanol, 1-pentanol, isoamylol, 1-hexanol, 1-heptanol, 1-octanol, propanoic acid, acetonitrile, and cyclohexanone from *T* = 274.05 to *T* = 323.05 K at 1 atm. Four thermodynamic phase equilibrium models (Apelblat equation, λh equation, NRTL model, and Wilson model) were used to correlate the experimental data. The relationship between the solubility of α-LM and the selected solvent parameters was analyzed by the KAT-LSER model. The dissolution enthalpy, entropy, and Gibbs free energy of α-LM in these solvents would be determined.

## 2. Experimental Section

### 2.1. Materials

The α-LM with a purity of 0.990 in the mass fraction was purchased from Sigma-Aldrich (St. Louis, MO, USA) and used without further purification, and the relevant information on the selected solvents has been listed in Table 1. All solvents were also used without further purification. The detailed information on the materials used in the present work was shown in Table 1.

### 2.2. Characterization

X-ray powder diffractometer (XRD) was used to verify the crystal forms of α-LM during the experiments, and the tests were carried out using a D-max 2000 VPC diffractometer with Cu Kα radiation (Rigaku, Tokyo, Japan). The diffraction angle is 5~60° with the scanning step of 0.02°. The characteristic peaks of XRD spectrum were located by using MDI Jade 6 software (Materials Data, Inc., Livermore, CA, USA).

The melting temperature (*T*_m_) and fusion entropy (Δ*_fus_H*) of α-LM were determined by a differential scanning calorimeter (DSC F204 Phoenix, Netzsch, Selb, Germany) under a nitrogen atmosphere. About 7 mg sample was put in a DSC pan and heated from 423.15 K to 533.15 K with a heating rate of 5 K/min. The measurement was repeated three times. The three replicate DSC measurements were taken from the purchased raw material samples and carried out separately.

### 2.3. Solubility Measurements

The solubility of α-LM was carried out in 15 mono-solvents (methanol, ethanol, 1-propanol, isopropanol, 1-butanol, 2-butanol, isobutanol, 1-pentanol, isoamylol, 1-hexanol, 1-heptanol, 1-octanol, propanoic acid, acetonitrile, and cyclohexanone) from 274.05 K to 323.05 K according to [19]. For the method, excess α-LM was added to a 250 mL jacketed glass vessel containing about 100 mL of the above solvent. Then, the mixture solution was continuously stirred by a magnetic agitator (HJ-1, Anhui Haixin, China) and the solute was added at regular intervals to ensure that there were always visible solids in the solution. The temperature was kept at a required value by the smart refrigerated and heating circulators with an accuracy of ±0.01 K (FP 51, JULABO, Seelbach, Germany). The actual solution temperature was measured by a calibrated mercury thermometer with an accuracy of ±0.01 K. The mass of α-LM dissolved in unit mass supernatant was measured every hour until the figure accounted to two decimal places maintained unchanged to find out the dissolution equilibrium time. The magnetic agitator was turned off and settled for 7 h at the same temperature and the residual solids were sampled for XRD measurement after drying. Subsequently, the supernatant was filtered and transferred to a pre-weighed weighing bottle (*m*_1_) by a preheated syringe equipped with a 0.45 μm filter. Then, the total weight of the bottle and solution was measured quickly (*m*_2_). The saturated solution was placed in a vacuum oven at 313.15 K until the weight of the sample was constant (*m*_3_). All the above samples were weighed by an analytical balance with an accuracy of ±0.0001 g (Mettler Toledo AL204, Nänikon, Switzerland). To ensure accuracy, all experiments were tested three times and the arithmetic average value was used to calculate the solubility of α-LM.

The mole fraction solubility (*x*_1_) of α-LM was calculated by Equation (1):

(1) $$x_{1}=\frac{m_{A}/M_{A}}{m_{A}/M_{A}+m_{B}/M_{B}}$$

where *m_A_* = *m*_3_ − *m*_1_, is the mass of α-LM; *m_B_* = *m*_2_ − *m*_3_ is the mass of the solvent; *M_A_* and *M_B_* are the molecular weights of α-LM and the solvent, respectively.

## 3. Thermodynamic Models

The theories of correlation phase equilibrium data mainly include empirical model, state equation model, and activity coefficient model. The state equation model is usually too complicated because of the choice of mixing rules, which requires a large number of thermodynamic parameters in the calculation process, and is seldom used in the actual process. Therefore, this work chose the practical empirical model (Apelblat equation and λh equation) and activity coefficient model (NRTL model and Wilson model) to correlate solubility, which were used to correctly predict the saturation concentration of solute under different conditions.

### 3.1. Apelblat Equation

The Apelblat equation is a semi-empirical model with three parameters, which can be used to describe the general relationship between solubility and temperature [20,21,22]. It can be expressed as Equation (2). The Apelblat equation is suitable for interpolation, but does not have the function of extrapolation. Because the model is simple and the number of model parameters is small, if the model is used to correlate and predict the system with a wide range of solubility values, the deviation of the results will increase.

(2) $$\ln x_{1}=A+B/(T/K)+C\ln(T/K)$$

where *x*_1_ is the mole fraction solubility of α-LM; *T* is the absolute temperature; *A*, *B*, and *C* are adjustable constants; *C* demonstrates the effect of temperature on the fusion enthalpy.

### 3.2. λh Equation

The *λh* equation is another empirical model with two parameters (λ and h) proposed by Buchowski et al. in 1981, which can be used to describe the relationship between solubility and temperature [23]. The model is suitable for studying the solvent activity along a saturation line and solubility of hydrogen-bonding solids [24], and the equation can be expressed as Equation (3). When dealing with multicomponent systems, two parameters are still maintained. Good results can also be obtained by treating mixed solvents as virtual unitary solvents. However, it is difficult to use the solubility data of pure solvents to predict the solubility behavior of multicomponent systems.

(3) $$\ln\left[1+\lambda \frac{1-x_{1}}{x_{1}}\right]=\lambda h(\frac{1}{T/K}-\frac{1}{T_{m}/K})$$

Herein, *x*_1_ is the mole fraction solubility of α-LM; *T* and *T_m_* are the absolute temperature and melting temperature of the solute, respectively; *λ* and *h* are the two parameters of the *λh* equation, which reflect the non-ideality of the solution and enthalpy of the solution, respectively.

### 3.3. NRTL Model

Based on the solid–liquid phase equilibrium theory proposed by Renon et al. and the concept of local composition, the NRTL model is used to describe the fluid phase equilibrium and calculate the activity coefficient of the solute [25,26,27]. Based on the activity coefficient model, the simplified equation can be described as Equation (4). The NRTL model can be applied to partially miscible and immiscible systems. Compared with other thermodynamic models, the regression parameters of NRTL model need a large amount of work, and sometimes the parameters can not be steadily extended from room temperature to a wider temperature range. When there is ionization equilibrium, the correlation of model parameters at different temperatures is more complex.

(4) $$\ln x_{1}=\frac{\Delta_{fus}H}{R}(\frac{1}{T_{m}/K}-\frac{1}{T/K})-\ln\gamma_{1}$$

Herein, *x*_1_ is the mole fraction solubility of α-LM; *γ*_1_ is the activity coefficient of solute; *T* is the experimental temperature; *T_m_* and Δ*_fus_H* are the melting temperature and fusion enthalpy of α-LM, respectively.

The *γ*_1_ can be calculated with the NRTL equation, which is expressed as Equations (5)–(8):

(5) $$\ln\gamma_{1}=x_{2}^{2}\left[\frac{\tau_{21}G_{21}^{2}}{{\left(x_{1}+x_{2}G_{21}\right)}^{2}}+\frac{\tau_{12}G_{12}^{}}{{\left(x_{2}+x_{1}G_{12}\right)}^{2}}\right]$$

(6) $$G_{ji}=\exp(-\alpha_{ji}\tau_{ji})$$

(7) $$\alpha_{ij}=\alpha_{ji}=\alpha$$

(8) $$\tau_{ij}=\frac{\Delta g_{ij}}{RT}$$

Herein, *γ*_1_ is the activity coefficient of component *i*; Δg*ij* is the Gibbs energy of intermolecular interaction; *α_ij_* is an adjustable constant, which usually varied from 0.2 to 0.47.

For model correlation, it can be assumed that the cross-interaction parameters between solvent and solute (*τ_ij_*) in the NRTL model have a linear relationship with temperature, which can be expressed as Equation (9) [28]:

(9) $$\tau_{ij}=a_{ij}+\frac{b_{ij}}{T/K}$$

Herein, *a_ij_* and *b_ij_* are equation parameters independent of composition and temperature.

### 3.4. Wilson Model

Based on the concept of excess free enthalpy *G^E^*, the Wilson model can describe the activity coefficient more concretely, which can be expressed as Equations (10)–(12) [29,30]. The model can reflect the effect of temperature on activity coefficient, has semi-theoretical physical meaning, and can predict the behavior of multicomponent systems. However, the Wilson model cannot be applied to the liquid phase stratification system, nor can it reflect the solution characteristics in which the activity coefficient has the highest or lowest value.

(10) $$\ln\gamma_{1}=-\ln(x_{1}+\Lambda_{12}x_{2})+x_{2}(\frac{\Lambda_{12}}{x_{1}+\Lambda_{12}x_{2}}-\frac{\Lambda_{21}}{x_{2}+\Lambda_{21}x_{1}})$$

(11) $$\Lambda_{12}=\frac{V_{2}}{V_{1}}\exp\left[-\frac{\lambda_{12}-\lambda_{11}}{RT}\right]=\frac{V_{2}}{V_{1}}\exp\left[-\frac{\Delta \lambda_{12}}{RT}\right]$$

(12) $$\Lambda_{21}=\frac{V_{1}}{V_{2}}\exp\left[-\frac{\lambda_{21}-\lambda_{22}}{RT}\right]=\frac{V_{1}}{V_{2}}\exp\left[-\frac{\Delta \lambda_{21}}{RT}\right]$$

Herein, *V*_1_ and *V*_2_ represent the molar volumes of α-LM and the solvents, respectively; *x*_1_ and *x*_2_ denote the mole fractions of α-LM and the solvent, respectively; Δ*λ_ij_* represents the energy parameters about cross-interactions between *i* and *j* components in the dissolution process; Λ*_ij_* represents the binary cross-interaction parameters in the Wilson model, which can be assumed to have a linear relationship with temperature [28]. The Wilson model can be expressed as Equation (13):

(13) $$\Lambda_{ij}=\frac{V_{j}}{V_{i}}\exp\left[-\left(a_{ij}+\frac{b_{ij}}{T/K}\right)\right]$$

Herein, *a_ij_* and *b_ij_* are the parameters of the model, which are independent of composition and temperature.

Finally, relative average deviation (*RAD*) and the root-mean square deviation (*RMSD*) were used to evaluate the overall correlation effects of the thermodynamic models. The values fitted by the model with smaller *RAD* or *RMSD* values mean that they are closer to the experimental values, implying a better model. The two criteria can be described as Equations (14) and (15):

(14) $$RAD=\frac{1}{N}\sum_{i}^{N}\left|\frac{x_{i}-x_{i}^{\mathrm{calc}}}{x_{i}}\right|$$

(15) $$RMSD={\left[\frac{\sum_{i=1}^{N}{\left(x_{i}^{\exp}-x_{i}^{\mathrm{calc}}\right)}^{2}}{N}\right]}^{1/2}$$

Herein, *N* represents the number of experimental values; *x_i_*^exp^ represents the experimental values; *x_i_*^calc^ represents the model fitted solubility values.

Parameters of four thermodynamic models for solubility correlation and KAT-LSER model regression were calculated by 1stOpt software (Professional Version 1.5) using the Levenberg–Marquardt and Universal Global Optimization (LM–UGO) method.

## 4. Results and Discussion

### 4.1. XRD Analysis

**Figure 2:**
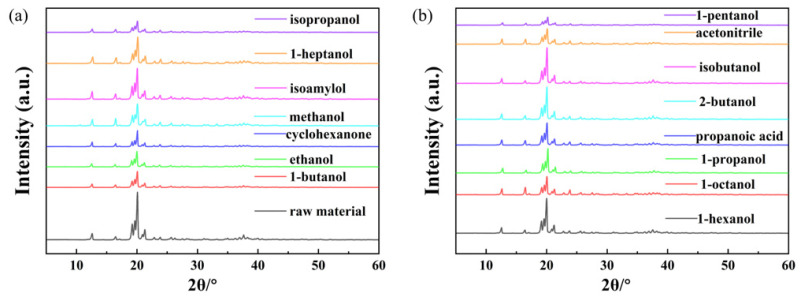
X-ray powder diffraction patterns of α-LM in different solvents: (**a**) the raw material, 1-butanol, ethanol, cyclohexanone, methanol, isoamylol, 1-heptanol, isopropanol; (**b**) 1-hexanol, 1-octanol, 1-propanol, propanoic acid, 2-butanol, isobutanol, acetonitrile, 1-pentanol.

Different crystal forms can lead to different solubility. The XRD patterns of α-LM raw material and residual solids of α-LM in the selected solvents are displayed in Figure 2. The characteristic peaks of α-LM raw material are located at 12.60°, 16.48°, 19.22°, 19.66°, 20.08°, 20.92°, 21.30°, 23.86°, 26.28°, and 37.64°. The residual α-LM solids in 15 solvents have the same crystal forms as the raw material, implying that there is no polycrystalline transformation of the solids in the dissolution process.

### 4.2. Melting Properties of α-LM

**Figure 3:**
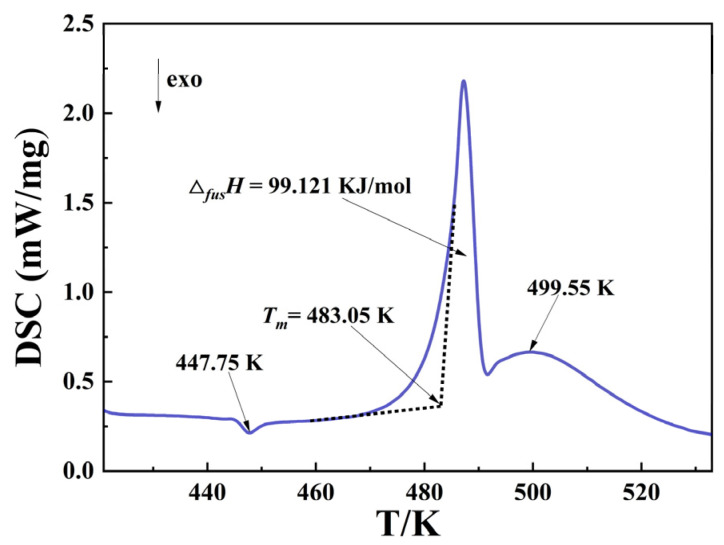
DSC plot of α-LM.

The melting temperature (*T_m_*) and fusion enthalpy (Δ*_fus_H*) were measured by DSC, and the results are shown in Figure 3. An exothermic peak appeared at 447.75 K, which was due to the transformation of amorphous lactose into crystals, and there is still a dehydration peak of α-LM near 418 K that has not been shown according to [31,32]. This is because during the heating process of DSC test, α-LM will preferentially lose the bound H_2_O molecule to become α-lactose. Therefore, the strong endothermic melting peak in DSC indicates that the melting of α-lactose at 483.05 K is not α-LM with a corresponding standard uncertainty of *u* (*T_m_*) = 0.5 K. The fusion enthalpy Δ*_fus_H* is 99.121 kJ/mol with a relative standard uncertainty of *u*_r_ (Δ*_fus_H*) = 0.05, and the fusion entropy (Δ*_fus_S*) of α-lactose is calculated as 205.20 J·mol^−1^·K^−1^ with Equation (16).

(16) $$\Delta_{fus}S=\frac{\Delta_{fus}H}{T_{m}/K}$$

In addition, a small exothermic peak was also observed at 499.55 K, which may be attributed to solid-state epimerization and melting of the sample powder caused by the heating and dehydration of α-LM, and about 29.1 ± 0.7% of the sample powder was converted into β-lactose at 463.15 K, and the results are following [32,33].

### 4.3. Solubility Data and Correlation

**Figure 4:**
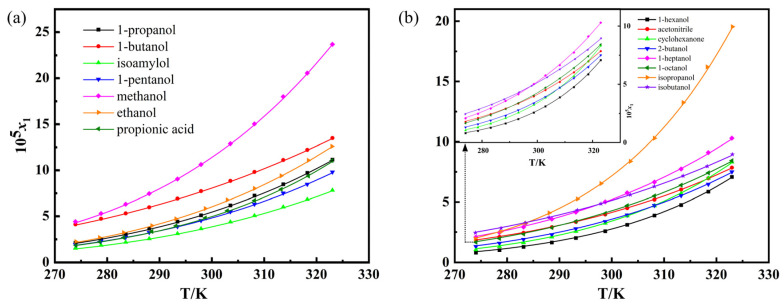
Solubility of α-LM in 15 solvents: (**a**) (*E*_T_(30) ≥ 49.0): 1-propanol, 1-butanol, isoamylol, 1-pentanol, methanol, ethanol, and propionic acid; (**b**) (*E*_T_(30) < 49.0): 1-hexanol, acetonitrile, cyclohexanone, 2-butanol, 1-heptanol, 1-octanol, isopropanol, and isobutanol. The trend lines were fitted with the Apelblat equation.

The experimental mole fraction solubility *x*_1_^exp^ of α-LM in 1-butanol, ethanol, cyclohexanone, methanol, isoamylol, 1-heptanol, and isopropanol, 1-hexanol, 1-octanol, 1-propanol, propanoic acid, 2-butanol, isobutanol, acetonitrile, and 1-pentanol from 274.05 K to 323.05 K are presented in Table S1. The relationship between temperature and solubility is shown in Figure 4. It can be seen that the solubility of α-LM increased with increasing temperature in the above solvents, implying that the dissolution process is endothermic. The α-LM has the largest solubility in methanol, but the smallest solubility in 1-hexanol.

To evaluate the differences of α-LM solubility in the given solvents, some physical parameters, including polarity index (*E*_T_ (30)), dipole moments (*μ*), dielectric constants (*ε*) and Hansen solubility parameters (*δ_H_*) are also shown in Table 2. Among them, *E*_T_(30) was also widely used to empirically evaluate the polarity of various molecular liquids and ionic liquids as an important parameter to describe the hydrogen bond and electrostatic interaction of solvents [34,35,36]. In this study, the solvents were divided into two groups according to the boundary of the values: *E*_T_(30) ≥ 49.0 and *E*_T_(30) < 49.0. It can be observed from Figure 4 that the solubility order of α-LM in the strong polar solvents is: methanol (*E*_T_(30) = 55.4) > 1-butanol (*E*_T_(30) = 49.7) > ethanol (*E*_T_(30) = 51.9) > 1-propanol (*E*_T_(30) = 50.7) > propionic acid (*E*_T_(30) = 55.0) > 1-pentanol (*E*_T_(30) = 49.1) > isoamylol (*E*_T_(30) = 49.0). In addition, the solubility of α-LM in weak polar solvents decreases in the sequence: isopropanol (above 283.55 K) > isobutanol > 1-heptanol > acetonitrile ≈ 1-octanol > 2-butanol > cyclohexanone > 1-hexanol below 298.15 K and isopropanol > 1-heptanol > isobutanol > 1-octanol > acetonitrile > cyclohexanone (below 318.45 K) ≈ 2-butanol > 1-hexanol above 298.15 K. In addition, the solubility order of α-LM in 15 pure solvents at 298.15 K is: methanol > 1-butanol > isopropanol > ethanol > 1-propanol > 1-heptanol > isobutanol > propionic acid > 1-pentanol > 1-octanol > acetonitrile > isoamylol > 2-butanol > cyclohexanone > 1-hexanol.

Based on the above results in Table S1 and Table 2, the solubility order of α-LM is not in accordance with the order of polarity of solvents, which deviates from the rule of “like dissolves like”. Therefore, the polarity of the solution is not a critical factor that affected the solubility of α-LM, so it is difficult to deduce the phenomenon by a single reason. The solubility of solute is significantly affected by solvent–solvent and solute–solvent interactions [37]. The α-LM contains 8 hydrogen receptor groups (-OH), and hydrogen bonds can be formed between the solute and some solvent molecules. The dissolution of α-LM in different polar solvents can cause various intermolecular forces. In addition, cohesive energy density has been used to describe the binding degree of solvent–solvent [38]. The exact mechanism which led to the complex solubility performance of α-LM is still not clear and more investigation should be performed.

The solubility data of α-LM in different solvents can offer useful information to optimize the crystallization process. In this work, four thermodynamic models were used to correlate the experimental data. The model parameters, including *RAD* and *RMSD*, are shown in Table 3, Table 4, Table 5 and Table 6. The average *RAD* values of the four models were 0.53% (Apelblat), 0.55% (Wilson), 1.11% (λh), and 0.34% (NRTL). The average values of *RAD* and *RMSD* of the NRTL model are the lowest among the four models, implying that this model is more suitable for the data correlation.

### 4.4. Solvent Effect

Kamlet et al. proposed the KAT-LSER model to analyze the solvent effect to describe complex solubility performance, which divided the intermolecular forces into non-specific ones and specific ones [42,43]. The KAT-LSER model can reasonably explain the effect of various physical properties of the solvent on the solute solubility through multiple linear regression correlation of solute solubility data in different pure solvents [44]. The expression of KAT-LSER model can be expressed as Equation (17):

(17) $$\ln x=c_{0}+c_{1}\alpha +c_{2}\beta +c_{3}\pi^{*}+c_{4}(\frac{V_{s}\delta_{H}^{2}}{100RT})$$

Herein, *α* and *β* are the acidity and basicity of hydrogen bonds, respectively; π* is the dipolarity-polarizability of the solvents; *δ_H_* is the Hansen solubility parameter of some solvents; *c*_0_ is a constant, depending on the solute; *c*_1_, *c*_2_, *c*_3_, and *c*_4_ are all constants, indicating the influence of the four property parameters of solvents on the solubility of α-LM; *V_s_* = 235.4967 cm^3^/mol is the molar volume of α-LM; *R* = 8.314 J/(mol·K) is the molar gas constant; *x* is the solubility of *α*-LM at *T*; *T* is the experimental temperature around 298.15 K.

The values of *α*, *β*, π* and *δ_H_* of fourteen solvents used in the correlation of KAT-LSER model are listed in Table 3. Multiple linear regression analysis was used to correlate the solubility data of α-LM with the property parameters of solvents according to Equation (17), and the correlation result is shown in Equation (18).

(18) $$\begin{array}{l} \ln x=-11.4635+0.2313\alpha +0.1419\beta +1.5897\pi^{*}+2.1243(\frac{V_{s}\delta_{H}^{2}}{100RT})\\ n=14,\;R^{2}=0.9980,\;RMSD=0.1965,\;RSS=0.5407,\;\chi^{2}=0.0191 \end{array}$$

Herein, *n* is the number of solvents used, except 1-heptanol. For the regression effect, *R*^2^ is the square of the correlation coefficient; *RMSD* is the root-mean square deviation; *RSS* is the residual sum of squares; *χ*^2^ is the value of the Chi-Squared test. These indicators show a reliable result.

In the fitted KAT-LSER model, *c*_0_ = −11.4635, indicating that the dissolution process of α-LM needs to overcome the strong crystal lattice cohesion energy, so it is difficult to dissolve. However, *c*_1,_ *c*_2,_ and *c*_3_ are all positive (*c*_1_ = 0.2313, *c*_2_ = 0.1419, *c*_3_ = 1.5897), implying that the solubility of α-LM increases with the rising of hydrogen bond donation ability, electron pair donation ability, and polarity of the solvents. Furthermore, the coefficient of *δ_H_* is positive (*c*_4_ = 2.1243), which indicates that the solvent hydrogen bonding cohesion (Hansen solubility parameter) is the most beneficial to the dissolution of α-LM. In the solvent effect distribution ratio, the four parameters *α*, *β*, π*, and *δ_H_* are 1.49%, 0.91%, 10.22%, and 13.66%, respectively. The total distribution proportion of the solvent effect is 26.28%. As a result, the properties of α-LM have a more significant effect than the properties of solvents, which can explain the low solubility of α-LM in this study.

### 4.5. Thermodynamic Properties of Dissolution

The calculation of the thermodynamics of the dissolution of α-LM in selected solvents will guide its production applications, and the dissolution thermodynamic properties were described by the changes of Gibbs energy (*G*), enthalpy (*S*), and entropy (*H*) of the α-LM in the dissolution process. Such as Δ*_dis_H* can help to determine the energy exchanged in the crystallization system, Δ*_dis_S* can help to determine the degree of confusion in the system, Δ*_dis_G* can help to determine the difficulty of spontaneity and driving force of the process, which can be described as four stages: heating, melting, cooling, and mixing [45,46], expressed as Equation (19).

(19) $$\Delta_{dis}M=x(\Delta_{heat}M+\Delta_{fus}M+\Delta_{cool}M)+\Delta_{mix}M$$

Herein, *x* is the mole fraction of α-LM in a pure solvent; *M* can be considered as Gibbs energy (*G*), entropy (*S*), and enthalpy (*H*). The values of *S* and *H* in the heating (*_heat_M*) and cooling (*_cool_M*) process are lower than those in the fusion (*_fus_M*) process, so they can be ignored in the calculation of dissolution properties [46].

According to the Lewis–Randall law, the real thermodynamic properties of mixed solutions were composed of ideal mixing properties and excess thermodynamic properties, which can be expressed by Equation (20) [47]:

(20) $$\Delta_{mix}M=M^{E}+\Delta_{mix}M^{id}$$

Herein, *M* can be *S*, *H*, and *G*, representing entropy, enthalpy, and Gibbs energy, respectively; *M^E^* represents the excess property; Δ*_mix_M^id^* is the thermodynamic property of mixing in the ideal solution, which can be calculated as Equations (21)–(23), respectively [19,48].

(21) $$\Delta_{mix}S^{id}=-R(x_{1}\ln x_{1}+x_{2}\ln x_{2})$$

(22) $$\Delta_{mix}H^{id}=0$$

(23) $$\Delta_{mix}G^{id}=RT(x_{1}\ln x_{1}+x_{2}\ln x_{2})$$

Herein, *x*_1_ and *x*_2_ are the mole fraction of α-LM and the solvent, respectively; Δ*_mix_S ^id^*, Δ*_mix_H* *^id^*, and Δ*_mix_G ^id^* refer to the mixing entropy, enthalpy, and Gibbs energy of the deal solution, respectively.

The excess mixing property (*S^E^*, *H^E^*, and *G^E^*) can be calculated as Equations (24)–(26) [49,50], based on the correlation results of the Wilson model.

(24) $$S^{E}=\frac{H^{E}-G^{E}}{T/K}$$

(25) $$H^{E}=-T^{2}\left[\frac{\partial \left(G^{E}/T\right)}{\partial T}\right]=Rx_{1}x_{2}\left(\frac{b_{12}\Lambda_{12}}{x_{1}+\Lambda_{12}x_{2}}+\frac{b_{21}\Lambda_{21}}{x_{2}+\Lambda_{21}x_{1}}\right)$$

(26) $$G^{E}=-RT(x_{1}\ln(x_{1}+x_{2}\Lambda_{12})+x_{2}\ln(x_{2}+x_{1}\Lambda_{21}))$$

**Figure 5:**
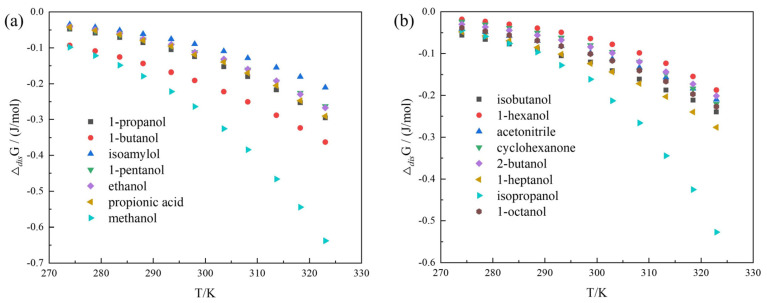
Δ*_dis_G* of α-LM in 15 solvents: (**a**) (*E*_T_(30) ≥ 49.0): 1-propanol, 1-butanol, isoamylol, 1-pentanol, methanol, ethanol, and propionic acid; (**b**) (*E*_T_(30) < 49.0): 1-hexanol, acetonitrile, cyclohexanone, 2-butanol, 1-heptanol, 1-octanol, isopropanol, and isobutanol.

Based on the experimental solubility data and the fitted parameters of the Wilson model, the values of dissolution properties (Δ*_dis_H*, Δ*_dis_S* and Δ*_dis_G*) of α-LM in 15 mono-solvents from 274.05 to 323.05 K were calculated and are listed in Table S2. Positive values of Δ*_dis_H* demonstrate that the dissolution of α-LM is endothermic in selected 15 mono-solvents, and the positive values of Δ*_dis_S* are the degree of confusion, indicating that the dissolution process of α-LM is entropy-driven. In addition, the higher the solubility of α-LM, the higher are the values of Δ*_dis_H* and Δ*_dis_S*, which indicates that the heat absorption and entropy increase are also increased during the dissolution of the corresponding solvent. The dissolution of α-LM in these 15 mono-solvents is driven by both heat and entropy. The Δ*_dis_G* of α-LM in 15 mono-solvents are all negative, and the higher the dissolution temperature is, the lower the value is and the greater the decline rate is, implying that the dissolution process of α-LM is spontaneous and favorable for high temperature. Moreover, Figure 5 shows the Δ*_dis_G* of α-LM in 15 solvents. By comparison with Figure 4, it can be seen that the decreasing order of Δ*_dis_G* values in different pure solvents with temperature is the same as that of their solubility increasing with temperature. This shows that both theoretical calculation and experiment section are reliable.

## 5. Conclusions

In this work, the solubility of α-LM in 15 pure solvents, namely methanol, ethanol, 1-propanol, isopropanol, 1-butanol, 2-butanol, isobutanol, 1-pentanol, isoamylol, 1-hexanol, 1-heptanol, 1-octanol, propanoic acid, acetonitrile, and cyclohexanone, was measured in the temperature range of 274.05 K to 323.05 K under atmospheric pressure by a static gravimetric method. The solubility of α-LM in all selected solvents increased with increasing temperature. At the given temperature, the solubility of α-LM in methanol was the highest and that in 1-hexanol was the lowest. Four thermodynamic models including the Apelblat equation, λh equation, NRTL model and Wilson model were used to correlate the experimental data. The fitting values of the above models were close to the experimental values, and the NRTL model provided the best fitting results. The solvent effect showed that hydrogen bond donation ability, electron pair donation ability, and polarity of the solvents were beneficial for the dissolution of α-LM. In addition, the dissolution thermodynamics (Δ*_dis_H*, Δ*_dis_S,* and Δ*_dis_G*) were calculated. The Δ*_dis_H* and Δ*_dis_S* were positive, but Δ*_dis_G* was negative, implying that the dissolution process was endothermic, entropy-driven, and spontaneous. The effect of the dissolution process of α-LM is important to its recrystallization and purification.

## Figures and Tables

**Table 1:** Detailed information about the materials used.

Chemicals	CAS No.	Source	Mass Fraction Purity	Analysis Method	Molar Volume ^c^ (cm^3^·mol^−1^)
α-LM	5989-81-1	Sigma-Aldrich, St. Louis, MO, USA	≥0.990	GC ^a^	235.4967
Methanol	67-56-1	Saan Chemical Technology (Shanghai, China) Co., Ltd.	≥0.995	GC ^a^	40.5057
Ethanol	64-17-5	Tianjin Kemao Chemical Reagent Co., Ltd. (Tianjin, China)	≥0.997	GC ^a^	58.3904
1-Propanol	71-23-8	Saan Chemical Technology (Shanghai) Co., Ltd. (Shanghai, China)	≥0.995	GC ^a^	75.1188
1-Butanol	71-36-3	Adamas-beta, Shanghai Titan Scientific Co., Ltd. (Shanghai, China)	≥0.995	GC ^a^	91.5062
Isobutanol	78-83-1	Saan Chemical Technology (Shanghai) Co., Ltd. (Shanghai, China)	≥0.990	GC ^a^	92.3064
2-Butanol	78-92-2	Saan Chemical Technology (Shanghai) Co., Ltd. (Shanghai, China)	≥0.990	GC ^a^	92.6525
1-Pentanol	71-41-0	Saan Chemical Technology (Shanghai) Co., Ltd. (Shanghai, China)	≥0.990	GC ^a^	106.2048
Isoamylol	123-51-3	Saan Chemical Technology (Shanghai) Co., Ltd. (Shanghai, China)	≥0.990	GC ^a^	108.8272
1-Hexanol	111-27-3	Saan Chemical Technology (Shanghai) Co., Ltd. (Shanghai, China)	≥0.980	GC ^a^	125.0610
1-Heptanol	111-70-6	Saan Chemical Technology (Shanghai) Co., Ltd. (Shanghai, China)	≥0.990	GC ^a^	141.3625
1-Octanol	111-87-5	Saan Chemical Technology (Shanghai) Co., Ltd. (Shanghai, China)	≥0.980	GC ^a^	158.0461
Propanoic Acid	79-09-4	Saan Chemical Technology (Shanghai) Co., Ltd. (Shanghai, China)	≥0.990	GC ^a^	74.8263
Acetonitrile	75-05-8	General-reagent, Shanghai Titan Scientific Co., Ltd. (Shanghai, China)	≥0.990	GC ^a^	52.2591
Cyclohexanone	108-94-1	Saan Chemical Technology (Shanghai) Co., Ltd. (Shanghai, China)	≥0.990	GC ^a^	102.9832
Isopropanol	67-63-0	Saan Chemical Technology (Shanghai) Co., Ltd. (Shanghai, China)	≥0.999	HPLC ^b^	76.4609

^a^ Gas chromatography. ^b^ High-performance liquid chromatography. Both the mass fraction purity and analysis method were provided by corresponding suppliers. ^c^ Molar volume equals molar mass divided by density. Both the molar mass and density were taken from [17,18].

**Table 2:** Some physical properties of selected solvents.

Solvent	*E*_T_(30) ^a^	*α* ^a^	*β* ^a^	π* ^a^	*μ* ^b^	*ε* ^c^	*δ_H_* ^d^
Methanol	55.40	0.98	0.66	0.60	1.70	32.60	22.30
Ethanol	51.90	0.86	0.75	0.54	1.70	22.40	19.40
1-Propanol	50.70	0.84	0.9	0.52	1.70	20.10	17.40
Isopropanol	48.40	0.76	0.84	0.48	1.66	18.30	16.40
1-Butanol	49.70	0.84	0.84	0.47	1.66	18.20	15.80
Isobutanol	48.60	0.79	0.84	0.40	1.70	17.70	15.90
2-Butanol	47.10	0.69	0.80	0.40	1.70	16.56	14.50
1-Pentanol	49.10	0.84	0.86	0.40	1.70	13.90	13.90
Isoamylol	49.00	0.84	0.86	0.40	1.80	15.20	13.30
Cyclohexanone	39.80	0.00	0.53	0.68	3.10	18.20	12.70
1-Hexanol	48.80	0.80	0.84	0.40	-	-	13.00
1-Heptanol	-	-	-	-	-	-	11.90
1-Octanol	48.10	0.77	0.81	0.40	1.90	-	6.10
Acetonitrile	45.60	0.19	0.40	0.66	3.20	37.50	5.10
Propinoic acid	55.00	1.12	0.45	0.58	-	-	12.40

^a^ Dimroth and Reichardt’s polarity parameter, Kamlet–Taft parameter. Taken from [39]. ^b^ Dipole moment, *μ/D*. Taken from [40]. ^c^ Dielectric constant at *T* = 293.15 K. Taken from [40]. ^d^ Hydrogen bonding cohesion (Hansen) solubility parameter, the unit is MPa ^1/2^. Taken from [41]. “-” means the data were not found.

**Table 3:** Parameters and deviations of Apelblat equation for the solubility of α-LM in 15 solvents.

Solvent	A	B	C	10^3^ *RAD*	10^6^ *RMSD*
Methanol	3.0766	−3158.8693	−0.2847	5.3031	0.6003
Ethanol	41.7699	−4985.8779	−6.1120	4.6061	0.2374
1-Propanol	26.4854	−4185.2840	−3.9175	4.1847	0.2885
Isopropanol	−27.8252	−2736.3574	4.8044	6.3433	0.6439
Acetonitrile	−27.5986	−1497.9216	3.9436	3.4318	0.1189
1-Butanol	4.5383	−2459.6923	−1.0101	2.9809	0.2425
Isobutanol	−6.8557	−2120.3304	0.7088	2.4968	0.1839
2-Butanol	4.0985	−3337.5824	−0.5650	4.9549	0.2350
1-Pentanol	17.0290	−3736.0487	−2.5440	5.6961	0.3223
Isoamylol	32.6382	−4461.5797	−4.8957	4.6822	0.1659
1-Hexanol	−47.5650	−1692.9922	7.4855	6.8966	0.2083
1-Heptanol	−2.2769	−2787.9947	0.2990	5.8348	0.3460
1-Octanol	28.3460	−4215.4499	−4.2710	3.7780	0.1634
Cyclohexanone	−27.1953	−2419.8664	4.3772	9.7513	0.3162
Propinoic acid	−43.2062	−1265.0271	6.5779	8.4350	0.4790
Average				5.2917	0.3034

**Table 4:** Parameters and deviations of λh equation for the solubility of α-LM in 15 solvents.

Solvent	10^3^λ	h	10^3^ *RAD*	10^6^ *RMSD*
Methanol	4.8545	615,583.7552	4.8535	0.7199
Ethanol	3.1697	997,826.5251	15.6423	1.1342
1-Propanol	2.3316	1,285,742.1272	13.0935	0.8522
Isopropanol	16.0734	267,512.2672	26.7948	1.4094
Acetonitrile	1.0672	2,444,208.9247	5.9051	0.2256
1-Butanol	0.8784	2,233,532.0823	6.6374	0.5483
Isobutanol	0.7550	2,895,562.0914	4.7101	0.2708
2-Butanol	1.7099	1,806,144.9113	5.6122	0.2595
1-Pentanol	1.8496	1,573,562.5892	7.2038	0.5004
Isoamylol	1.4327	2,013,266.8911	8.6628	0.3579
1-Hexanol	4.2654	940,265.8582	18.1818	0.3672
1-Heptanol	1.6977	1,641,263.9352	6.9722	0.3709
1-Octanol	1.4556	1,941,581.3104	9.2550	0.3589
Cyclohexanone	3.9075	965,202.1135	14.0863	0.4350
Propinoic acid	2.7888	1,145,606.1334	5.3089	0.5203
Average			10.1946	0.5554

**Table 5:** Parameters and deviations of Wilson model for the solubility of α-LM in 15 solvents.

Solvent	*a*_12_	*b*_12_	*a*_21_	*b*_21_	10^3^ *RAD*	10^6^ *RMSD*
Methanol	24.4427	−8960.6721	0.4448	34.8474	5.4478	0.6006
Ethanol	25.7710	−8790.9862	0.0104	−0.0075	7.8584	0.4502
1-Propanol	26.3558	−8924.0253	−0.2396	−0.0038	5.5948	0.3651
Isopropanol	17.2970	−7415.4742	7.5370	−1468.9739	6.9079	0.6353
Acetonitrile	28.5466	−8231.7192	−0.2444	−119.7887	2.6844	0.1094
1-Butanol	28.1475	−9769.2383	−0.2111	1.1678	3.2012	0.2580
isobutanol	32.7860	−9600.4299	−1.1306	−0.0058	2.9048	0.1557
2-Butanol	26.4039	−8729.5396	−0.4449	−6.1837	4.7570	0.2359
1-Pentanol	26.8768	−8963.5924	−0.5753	1.8416	4.7443	0.3470
Isoamylol	26.5113	−8946.4676	−0.4400	0.0851	5.5650	0.2588
1-Hexanol	25.6327	−6718.4932	−1.1445	−139.6036	5.7548	0.1595
1-Heptanol	31.3149	−9079.4249	−1.5496	3.4011	5.7054	0.3349
1-Octanol	27.4758	−9001.5085	−0.9517	0.0247	6.3363	0.2515
Cyclohexanone	30.4941	−8406.5104	−1.4374	17.9929	9.1296	0.2281
Propinoic acid	32.0520	−9018.3050	−1.1804	29.1649	6.0851	0.3158
Average					5.5118	0.3137

**Table 6:** Parameters and deviations of NRTL model for the solubility of α-LM in 15 solvents.

Solvent	*α*	*a*_12_	*b*_12_	*a*_21_	*b*_21_	10^3^ *RAD*	10^6^ *RMSD*
Methanol	7.6935	−38.6535	12,490.0130	23.4391	−8826.6631	0.2460	0.4874
Ethanol	−0.3667	2.8759	−920.7980	21.4235	−7978.7635	4.6061	0.2389
1-Propanol	17.3089	−4.9224	1601.6066	24.3282	−8873.3037	4.0046	0.2632
Isopropanol	1.3652	0.1969	−418.1983	−9.4994	5151.6911	3.2121	0.1504
Acetonitrile	44.7979	−0.2714	84.6165	25.9768	−9283.6565	2.6844	0.1036
1-Butanol	10.5622	−27.8827	9009.6038	26.8760	−9754.0188	2.6124	0.2014
Isobutanol	21.3971	−12.1041	3908.5777	26.7979	−9593.3165	1.8288	0.0764
2-Butanol	−0.0345	−2.9422	872.3605	27.3048	−9624.3411	4.9549	0.2351
1-Pentanol	4.3661	−59.3899	19,189.7345	24.6120	−8920.9197	3.9488	0.1798
Isoamylol	23.2447	−4.4141	1437.1100	24.8075	−8912.0932	2.3763	0.1222
1-Hexanol	35.1965	−1.5530	499.1438	21.9911	−7964.3999	4.7214	0.1074
1-Heptanol	11.0309	−2.0698	688.8884	24.7314	−8983.8417	4.6317	0.3104
1-Octanol	−0.1241	−4.5717	1289.6954	29.2548	−10,195.5144	3.7780	0.1624
Cyclohexanone	7.7270	−3.9109	1279.8161	22.1958	−8097.0602	4.2176	0.1130
Propinoic acid	32.1107	−13.0524	4210.3300	23.8667	−8708.3199	2.9581	0.1232
Average						3.3854	0.1917

## Data Availability

Not applicable.

## Supplementary Materials

The following supporting information can be downloaded at: https://www.mdpi.com/article/10.3390/pharmaceutics14091774/s1, Table S1: Experimental and Model Fitted Solubility of α-LM in 15 Solvents (P = 0.1 MPa); Table S2: Dissolution Thermodynamic Properties of α-LM in 15 Solvents.
