# Supplementary material for: Crystallization Thermodynamics of α-Lactose Monohydrate in Different Solvents
Source: Pharmaceutics. 2022 Aug 25;14(9):1774. doi: 10.3390/pharmaceutics14091774 (PMC9506588; doi:10.3390/pharmaceutics14091774)
Supplement: Supplementary file 1 [file pharmaceutics-14-01774-s001.zip › pharmaceutics-1864968-supplementary.pdf]

Supporting Information

Crystallization Thermodynamics of α-lactose Monohydrate in Different Solvents

Youliang Guan [1], Zujin Yang [1*], Kui Wu [2] and Hongbing Ji [3*]

[1]School of Chemical Engineering and Technology, Sun Yat-sen University, Zhuhai 519082, China
[2]School of Chemistry and Chemical Engineering, Jinggangshan University, Ji'an 343009, China
[3]School of Chemistry, Sun Yat-sen University, Guangzhou 510275, China; jihb@mail.sysu.edu.cn

*Correspondence: yangzj3@mail.sysu.edu.cn; jihb@mail.sysu.edu.cn

Table S1. Experimental and Model Fitted Solubility of α-LM in 15 Solvents (P = 0.1 MPa) [a].

T/K	$10^5x_1^{exp}$	$10^5x_1^{Apelblat}$	$10^5x_1^{\lambda h}$	$10^5x_1^{NRTL}$	$10^5x_1^{Wilson}$
			Methanol		
273.95	4.39	4.31	4.36	4.39	4.31
278.85	5.28	5.25	5.29	5.28	5.25
283.55	6.28	6.30	6.33	6.28	6.31
288.05	7.43	7.47	7.48	7.43	7.47
293.45	9.02	9.09	9.08	9.02	9.09
297.95	10.60	10.65	10.63	10.60	10.65
303.55	12.86	12.88	12.84	12.86	12.88
308.15	15.01	14.98	14.94	15.02	14.98
313.65	17.98	17.84	17.81	17.97	17.84
318.25	20.53	20.55	20.55	20.53	20.55
323.05	23.67	23.72	23.77	23.67	23.72
			Ethanol		
274.05	2.21	2.18	2.16	2.18	2.24
278.55	2.66	2.65	2.61	2.65	2.70
283.15	3.20	3.21	3.15	3.21	3.24
288.55	3.96	3.98	3.89	3.98	3.98
293.15	4.69	4.73	4.63	4.73	4.72
298.75	5.81	5.80	5.68	5.80	5.77
302.95	6.75	6.71	6.60	6.71	6.67
308.15	7.99	7.98	7.90	7.98	7.94
313.25	9.37	9.40	9.38	9.40	9.37
318.45	11.05	11.02	11.12	11.02	11.02
322.95	12.57	12.58	12.84	12.58	12.64
			1-Propanol		
273.95	2.10	2.08	2.06	2.09	2.12
278.85	2.52	2.54	2.50	2.54	2.56

283.55	3.04	3.05	3.00	3.04	3.06
288.05	3.63	3.61	3.54	3.60	3.61
293.45	4.39	4.38	4.31	4.38	4.38
297.95	5.08	5.12	5.04	5.12	5.11
303.55	6.15	6.17	6.10	6.18	6.15
308.15	7.22	7.15	7.09	7.17	7.12
313.65	8.45	8.46	8.46	8.48	8.45
318.25	9.68	9.70	9.77	9.67	9.70
323.05	11.12	11.12	11.30	11.12	11.15
			Isopropanol		
273.95	1.93	1.95	1.80	1.95	1.96
278.85	2.54	2.53	2.38	2.53	2.53
283.55	3.23	3.22	3.07	3.22	3.22
288.05	4.10	4.04	3.89	4.07	4.04
293.45	5.25	5.26	5.13	5.26	5.26
297.95	6.53	6.52	6.40	6.55	6.51
303.55	8.37	8.44	8.36	8.39	8.44
308.15	10.29	10.38	10.34	10.28	10.38
313.65	13.25	13.21	13.23	13.23	13.21
318.25	16.21	16.07	16.16	16.21	16.07
323.05	19.54	19.62	19.80	19.54	19.61
			1-Butanol		
273.95	4.09	4.07	4.14	4.07	4.08
278.85	4.71	4.68	4.73	4.68	4.69
283.55	5.29	5.32	5.35	5.32	5.33
288.05	5.96	6.00	6.00	6.00	6.00
293.45	6.89	6.89	6.86	6.89	6.89
297.95	7.70	7.70	7.66	7.70	7.69
303.55	8.82	8.80	8.75	8.81	8.79

308.15	9.79	9.78	9.73	9.80	9.77
313.65	11.09	11.06	11.02	11.08	11.05
318.25	12.19	12.20	12.21	12.19	12.20
323.05	13.47	13.48	13.56	13.47	13.49
			Isobutanol		
273.95	2.44	2.45	2.46	2.45	2.45
278.85	2.83	2.84	2.85	2.84	2.85
283.55	3.26	3.26	3.26	3.26	3.27
288.05	3.72	3.71	3.70	3.71	3.71
293.45	4.32	4.30	4.28	4.31	4.30
297.95	4.86	4.85	4.83	4.85	4.85
303.55	5.60	5.60	5.58	5.61	5.60
308.15	6.29	6.29	6.27	6.29	6.28
313.65	7.17	7.18	7.17	7.18	7.17
318.25	7.96	8.00	8.02	7.96	8.00
323.05	8.96	8.93	8.98	8.96	8.94
			2-Butanol		
274.05	1.30	1.30	1.31	1.30	1.30
278.55	1.57	1.57	1.58	1.57	1.57
283.15	1.89	1.89	1.89	1.89	1.89
288.55	2.29	2.33	2.33	2.33	2.33
293.15	2.78	2.76	2.76	2.76	2.76
298.75	3.37	3.39	3.38	3.39	3.38
302.95	3.92	3.92	3.91	3.92	3.92
308.15	4.72	4.68	4.66	4.68	4.67
313.25	5.52	5.53	5.52	5.53	5.53
318.45	6.47	6.52	6.52	6.52	6.52
322.95	7.51	7.48	7.50	7.48	7.49
			1-Pentanol		

273.95	1.89	1.87	1.88	1.87	1.89
278.85	2.28	2.27	2.27	2.27	2.28
283.55	2.69	2.72	2.71	2.71	2.73
288.05	3.21	3.21	3.18	3.20	3.21
293.45	3.85	3.88	3.85	3.87	3.88
297.95	4.51	4.53	4.49	4.52	4.52
303.55	5.48	5.44	5.40	5.45	5.43
308.15	6.29	6.29	6.26	6.31	6.28
313.65	7.49	7.44	7.43	7.48	7.43
318.25	8.45	8.52	8.54	8.45	8.52
323.05	9.78	9.76	9.85	9.78	9.78
			Isoamylol		
273.95	1.49	1.47	1.52	1.49	1.50
278.85	1.81	1.80	1.83	1.81	1.82
283.55	2.15	2.16	2.18	2.16	2.17
288.05	2.53	2.55	2.56	2.55	2.56
293.45	3.08	3.10	3.09	3.09	3.09
297.95	3.62	3.62	3.60	3.61	3.60
303.55	4.35	4.36	4.32	4.35	4.33
308.15	5.05	5.04	5.00	5.04	5.02
313.65	5.99	5.96	5.93	5.97	5.94
318.25	6.80	6.81	6.81	6.81	6.81
323.05	7.79	7.80	7.85	7.79	7.83
			1-Hexanol		
274.05	0.80	0.81	0.76	0.79	0.81
278.55	0.99	1.01	0.96	0.99	1.01
283.15	1.27	1.26	1.22	1.25	1.26
288.55	1.63	1.62	1.59	1.63	1.63
293.15	2.01	2.00	1.98	2.02	2.01

298.75	2.57	2.57	2.56	2.59	2.58
302.95	3.11	3.09	3.09	3.11	3.09
308.15	3.88	3.86	3.87	3.87	3.85
313.25	4.75	4.77	4.79	4.75	4.76
318.45	5.85	5.89	5.92	5.85	5.88
322.95	7.08	7.05	7.07	7.08	7.07
			1-Heptanol		
274.05	2.08	2.10	2.11	2.09	2.10
278.55	2.47	2.48	2.49	2.48	2.49
283.15	2.93	2.94	2.94	2.94	2.94
288.55	3.58	3.55	3.55	3.56	3.56
293.15	4.16	4.15	4.14	4.16	4.16
298.75	5.01	4.99	4.98	5.00	4.99
302.95	5.76	5.71	5.69	5.71	5.70
308.15	6.65	6.70	6.68	6.69	6.69
313.25	7.73	7.80	7.79	7.78	7.79
318.45	9.10	9.06	9.07	9.05	9.06
322.95	10.29	10.28	10.32	10.31	10.30
			1-Octanol		
274.05	1.66	1.65	1.70	1.65	1.68
278.55	1.97	1.98	2.01	1.98	2.00
283.15	2.34	2.36	2.38	2.36	2.37
288.55	2.88	2.87	2.88	2.87	2.88
293.15	3.39	3.38	3.36	3.38	3.37
298.75	4.05	4.08	4.05	4.08	4.06
302.95	4.70	4.67	4.64	4.67	4.65
308.15	5.50	5.49	5.46	5.49	5.47
313.25	6.39	6.40	6.37	6.40	6.38
318.45	7.41	7.43	7.44	7.43	7.43

322.95	8.43	8.42	8.47	8.42	8.45
		Cyclohexanone			
274.05	1.03	1.06	1.02	1.04	1.06
278.55	1.29	1.31	1.27	1.30	1.32
283.15	1.61	1.62	1.58	1.62	1.63
288.55	2.09	2.07	2.03	2.08	2.08
293.15	2.56	2.53	2.50	2.55	2.54
298.75	3.24	3.21	3.19	3.23	3.21
302.95	3.85	3.82	3.80	3.83	3.82
308.15	4.69	4.70	4.70	4.70	4.69
313.25	5.69	5.74	5.76	5.71	5.72
318.45	6.97	7.00	7.03	6.96	6.98
322.95	8.32	8.28	8.32	8.32	8.31
		Acetonitrile			
274.05	1.77	1.80	1.77	1.79	1.79
278.55	2.10	2.09	2.06	2.09	2.09
283.15	2.43	2.43	2.41	2.44	2.44
288.55	2.91	2.90	2.88	2.90	2.90
293.15	3.35	3.34	3.33	3.35	3.35
298.75	3.97	3.96	3.96	3.97	3.97
302.95	4.49	4.49	4.49	4.49	4.49
308.15	5.20	5.22	5.22	5.22	5.22
313.25	6.02	6.03	6.04	6.02	6.02
318.45	6.97	6.96	6.98	6.95	6.95
322.95	7.85	7.85	7.88	7.86	7.86
		Propinoic acid			
274.05	1.81	1.85	1.81	1.82	1.84
278.55	2.19	2.22	2.19	2.20	2.22
283.15	2.65	2.66	2.64	2.66	2.67

288.55	3.31	3.27	3.27	3.28	3.29
293.15	3.93	3.89	3.90	3.91	3.91
298.75	4.79	4.78	4.80	4.80	4.79
302.95	5.57	5.55	5.58	5.58	5.55
308.15	6.68	6.66	6.69	6.67	6.64
313.25	7.90	7.94	7.95	7.91	7.90
318.45	9.34	9.45	9.44	9.34	9.41
322.95	11.03	10.95	10.92	11.03	11.00

[a] x_1^{exp} is the experimental solubility of α-LM in pure solvents; the standard uncertainty of temperature is $u(T)$= 0.05 K; the relative uncertainty of pressure is $u_r(P)$ =0.06. The relative standard uncertainty of solubility is $u_r(x_1)$ =0.045.

Table S2. Dissolution Thermodynamic Properties of α-LM in 15 Solvents [a].

T (K)	$\Delta_{dis}H$ (J/mol)	$\Delta_{dis}S$ (J/mol/K)	$\Delta_{dis}G$ (J/mol)
	Methanol		
273.95	1.1224	0.0045	-0.0982
278.85	1.3499	0.0053	-0.1218
283.55	1.6074	0.0062	-0.1487
288.05	1.9007	0.0072	-0.1790
293.45	2.3075	0.0086	-0.2219
297.95	2.7112	0.0100	-0.2640
303.55	3.2903	0.0119	-0.3253
308.15	3.8414	0.0137	-0.3841
313.65	4.6004	0.0162	-0.4657
318.25	5.2538	0.0182	-0.5445
323.05	6.0575	0.0207	-0.6380
	Ethanol		
274.05	0.5760	0.0022	-0.0403
278.55	0.6918	0.0027	-0.0493

283.15	0.8326	0.0032	-0.0601
288.55	1.0298	0.0038	-0.0752
293.15	1.2216	0.0045	-0.0906
298.75	1.5124	0.0054	-0.1124
302.95	1.7569	0.0062	-0.1316
308.15	2.0810	0.0073	-0.1596
313.25	2.4393	0.0084	-0.1918
318.45	2.8761	0.0098	-0.2294
322.95	3.2734	0.0110	-0.2673
	1-Propanol		
273.95	0.5227	0.0021	-0.0475
278.85	0.6290	0.0025	-0.0586
283.55	0.7586	0.0029	-0.0712
288.05	0.9036	0.0034	-0.0853
293.45	1.0952	0.0041	-0.1053
297.95	1.2674	0.0047	-0.1247
303.55	1.5338	0.0056	-0.1530
308.15	1.7993	0.0064	-0.1799
313.65	2.1072	0.0074	-0.2172
318.25	2.4145	0.0084	-0.2530
323.05	2.7739	0.0095	-0.2954
	Isopropanol		
273.95	0.6394	0.0025	-0.0446
278.85	0.8535	0.0033	-0.0587
283.55	1.0936	0.0041	-0.0760
288.05	1.4004	0.0052	-0.0967
293.45	1.8094	0.0066	-0.1283
297.95	2.2665	0.0081	-0.1613
303.55	2.9241	0.0103	-0.2130

308.15	3.6123	0.0126	-0.2660
313.65	4.6771	0.0160	-0.3445
318.25	5.7435	0.0194	-0.4253
323.05	6.9502	0.0231	-0.5269
	Acetonitrile		
274.05	0.3871	0.0016	-0.0408
278.55	0.4596	0.0018	-0.0484
283.15	0.5341	0.0021	-0.0574
288.55	0.6398	0.0025	-0.0697
293.15	0.7405	0.0028	-0.0818
298.75	0.8798	0.0033	-0.0988
302.95	0.9990	0.0037	-0.1133
308.15	1.1604	0.0042	-0.1338
313.25	1.3532	0.0048	-0.1568
318.45	1.5785	0.0055	-0.1837
322.95	1.7971	0.0062	-0.2101
	1-Butanol		
273.95	0.7342	0.0030	-0.0930
278.85	0.8441	0.0034	-0.1087
283.55	0.9482	0.0038	-0.1257
288.05	1.0679	0.0042	-0.1438
293.45	1.2357	0.0048	-0.1681
297.95	1.3810	0.0053	-0.1907
303.55	1.5811	0.0059	-0.2220
308.15	1.7550	0.0065	-0.2506
313.65	1.9880	0.0073	-0.2884
318.25	2.1858	0.0079	-0.3232
323.05	2.4155	0.0086	-0.3628
	Isobutanol		

273.95	0.4711	0.0019	-0.0559
278.85	0.5466	0.0022	-0.0661
283.55	0.6293	0.0025	-0.0771
288.05	0.7183	0.0028	-0.0890
293.45	0.8350	0.0032	-0.1052
297.95	0.9386	0.0036	-0.1203
303.55	1.0824	0.0040	-0.1415
308.15	1.2175	0.0045	-0.1611
313.65	1.3926	0.0050	-0.1872
318.25	1.5547	0.0056	-0.2116
323.05	1.7637	0.0062	-0.2398
2-Butanol			
274.05	0.3433	0.0014	-0.0297
278.55	0.4140	0.0016	-0.0363
283.15	0.4976	0.0019	-0.0444
288.55	0.6032	0.0023	-0.0558
293.15	0.7321	0.0027	-0.0674
298.75	0.8885	0.0033	-0.0841
302.95	1.0334	0.0037	-0.0988
308.15	1.2425	0.0044	-0.1198
313.25	1.4550	0.0051	-0.1440
318.45	1.7045	0.0059	-0.1726
322.95	1.9773	0.0067	-0.2011
1-Pentanol			
273.95	0.4665	0.0019	-0.0430
278.85	0.5626	0.0022	-0.0530
283.55	0.6644	0.0026	-0.0643
288.05	0.7926	0.0030	-0.0769
293.45	0.9498	0.0036	-0.0947

297.95	1.1132	0.0041	-0.1119
303.55	1.3509	0.0049	-0.1370
308.15	1.5515	0.0056	-0.1609
313.65	1.8482	0.0065	-0.1939
318.25	2.0847	0.0073	-0.2256
323.05	2.4118	0.0083	-0.2629
Isoamylol			
273.95	0.3691	0.0015	-0.0342
278.85	0.4486	0.0018	-0.0422
283.55	0.5321	0.0021	-0.0512
288.05	0.6259	0.0024	-0.0613
293.45	0.7618	0.0029	-0.0755
297.95	0.8950	0.0033	-0.0893
303.55	1.0758	0.0039	-0.1094
308.15	1.2483	0.0045	-0.1285
313.65	1.4810	0.0052	-0.1550
318.25	1.6838	0.0059	-0.1804
323.05	1.9286	0.0066	-0.2104
1-Hexanol			
274.05	0.2551	0.0010	-0.0184
278.55	0.3171	0.0012	-0.0234
283.15	0.4070	0.0015	-0.0297
288.55	0.5248	0.0020	-0.0391
293.15	0.6482	0.0024	-0.0490
298.75	0.8326	0.0030	-0.0641
302.95	1.0090	0.0036	-0.0779
308.15	1.2675	0.0044	-0.0986
313.25	1.5605	0.0054	-0.1234
318.45	1.9425	0.0066	-0.1545

322.95	2.3791	0.0079	-0.1872
	1-Heptanol		
274.05	0.4967	0.0020	-0.0479
278.55	0.5885	0.0023	-0.0577
283.15	0.6980	0.0027	-0.0693
288.55	0.8544	0.0033	-0.0854
293.15	0.9919	0.0037	-0.1014
298.75	1.1966	0.0044	-0.1241
302.95	1.3751	0.0050	-0.1438
308.15	1.5896	0.0057	-0.1716
313.25	1.8527	0.0066	-0.2031
318.45	2.1889	0.0076	-0.2399
322.95	2.4881	0.0086	-0.2762
	1-Octanol		
274.05	0.4025	0.0016	-0.0384
278.55	0.4783	0.0019	-0.0463
283.15	0.5692	0.0022	-0.0558
288.55	0.6991	0.0027	-0.0690
293.15	0.8226	0.0031	-0.0822
298.75	0.9840	0.0036	-0.1009
302.95	1.1418	0.0042	-0.1172
308.15	1.3347	0.0048	-0.1403
313.25	1.5526	0.0055	-0.1664
318.45	1.8005	0.0063	-0.1969
322.95	2.0483	0.0070	-0.2269
	Cyclohexanone		
274.05	0.3162	0.0012	-0.0242
278.55	0.3939	0.0015	-0.0305
283.15	0.4936	0.0019	-0.0384

288.55	0.6403	0.0024	-0.0499
293.15	0.7847	0.0029	-0.0620
298.75	0.9917	0.0036	-0.0799
302.95	1.1820	0.0042	-0.0961
308.15	1.4425	0.0051	-0.1201
313.25	1.7606	0.0061	-0.1486
318.45	2.1789	0.0074	-0.1838
322.95	2.6402	0.0089	-0.2203
	Propinoic acid		
274.05	0.4781	0.0019	-0.0419
278.55	0.5781	0.0023	-0.0514
283.15	0.7003	0.0027	-0.0628
288.55	0.8749	0.0033	-0.0790
293.15	1.0381	0.0039	-0.0953
298.75	1.2680	0.0046	-0.1190
302.95	1.4781	0.0053	-0.1399
308.15	1.7815	0.0063	-0.1701
313.25	2.1242	0.0074	-0.2050
318.45	2.5521	0.0088	-0.2472
322.95	3.0870	0.0105	-0.2904

[a] The values of $\Delta_{dis}G$, $\Delta_{dis}H$, and $\Delta_{dis}S$ were calculated with Equations (19)–(26).
